# Registro Brasileiro de Transplantes da ABTO e Registro de Transplante Cardíaco da ISHLT: Uma Valiosa Parceria

**DOI:** 10.36660/abc.20240370

**Published:** 2025-02-04

**Authors:** Fernando A. Atik, Paulo Manoel Pego-Fernandes, Juan A. Mejia, Livia A. Goldraich, Fabiana G. Marcondes-Braga, Estela Azeka, Fernando Augusto Figueira, Valter Duro Garcia, Luciana Haddad, Wida Cherikh, Josef Stehlik, Rebecca Cogswell

**Affiliations:** 1 Universidade de Brasília Brasília DF Brasil Universidade de Brasília, Brasília, DF – Brasil; 2 Hospital das Clínicas da Faculdade de Medicina da Universidade de São Paulo Instituto do Coração São Paulo SP Brasil Instituto do Coração do Hospital das Clínicas da Faculdade de Medicina da Universidade de São Paulo, São Paulo, SP – Brasil; 3 Hospital de Messejana Fortaleza CE Brasil Hospital de Messejana, Fortaleza, CE – Brasil; 4 Hospital de Clínicas de Porto Alegre Serviço de Cardiologia Porto Alegre RS Brasil Hospital de Clínicas de Porto Alegre – Serviço de Cardiologia, Porto Alegre, RS – Brasil; 5 Instituto de Medicina Integral Professor Fernando Figueira Recife PE Brasil Instituto de Medicina Integral Professor Fernando Figueira – Cardiologia Adulto, Recife, PE – Brasil; 6 Santa Casa de Misericórdia de Porto Alegre Porto Alegre RS Brasil Santa Casa de Misericórdia de Porto Alegre, Porto Alegre, RS – Brasil; 7 United Network for Organ Sharing Richmond Virginia EUA United Network for Organ Sharing, Richmond, Virginia – EUA; 8 University of Utah School of Medicine Division of Cardiovascular Medicine Salt Lake City Utah EUA The University of Utah School of Medicine – Division of Cardiovascular Medicine, Salt Lake City, Utah – EUA; 9 University of Minnesota Department of Medicine Division of Cardiology Minneapolis Minnesota EUA University of Minnesota – Department of Medicine, Division of Cardiology Minneapolis, Minnesota – EUA

**Keywords:** Registros, Transplante, Transplante de Coração

Desde 1983, dados de mais de 220 000 receptores de transplante de coração, pulmão, e coração e pulmão de 481 centros de transplante cardíaco e 260 centros de transplante pulmonar foram submetidos ao registro do ISHLT (*International Society for Heart and Lung Transplantation*). A missão do registro do ISHLT é melhorar o cuidado do paciente com doença cardíaca e pulmonar avançada. O registro tem atraído membros de diversas regiões, fornecido dados de transplantes torácicos de todo o mundo e catalisado colaborações clínicas e científicas. Dez hospitais brasileiros participavam do registro ISHL no passado e, durante esse período, foram observados alguns problemas. Primeiramente, havia um comprometimento heterogêneo dos centros participantes para completar o conjunto de dados. Segundo, havia uma escassez de recursos humanos em alguns hospitais dedicados ao registro. Terceiro, a coleta de dados era responsabilidade de cada centro, e a submissão dos dados (mediante pagamento) ao registro ISHLT não era obrigatória.^1^

Em 2021, o registro ISHLT foi interrompido devido aos novos regulamentos de compartilhamento de dados e novas abordagens de coleta de dados. Os campos e o processo de carregamento dos dados foram atualizados. Em 2024, o registro ISHLT começou a trocar dados somente com registros nacionais e regionais, tais como o *United Network for Organ Sharing* e o *Eurotransplant*, e não mais com hospitais ou instituições individuais.

**Figura 1:**
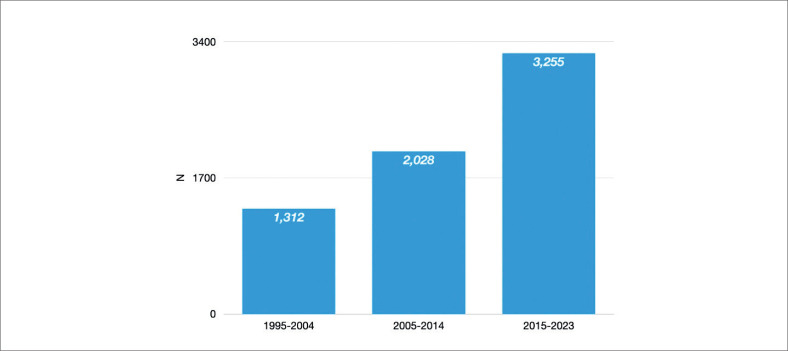
Número de transplantes cardíacos no Brasil por década entre 1995 e 2023.

**Figura 2:**
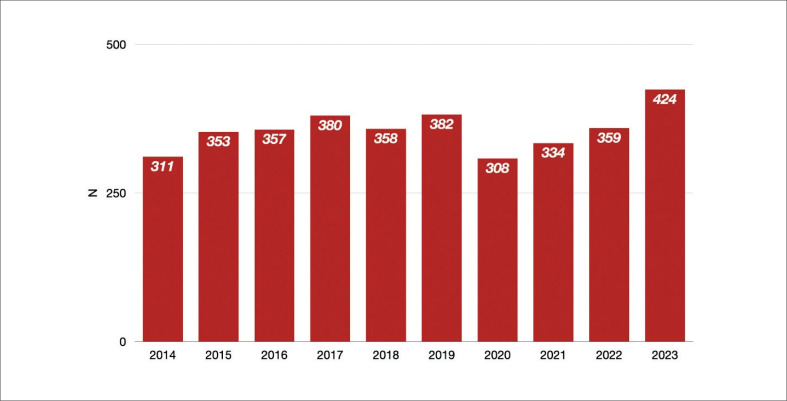
Número de transplantes cardíacos realizados no Brasil anualmente entre 2014 e 2023.

Para o Brasil continuar sua participação no novo formato do registro ISHLT, era compreensível que a Associação Brasileira de Transplante de Órgãos (ABTO) fosse escolhida como a sociedade nacional representante por já possuir seu próprio registro, o Registro Brasileiro de Transplantes (RBT). O RBT foi iniciado em 1996, e se trata do relatório oficial de todos os transplantes realizados no Brasil, incluindo transplantes de rim, coração, pâncreas, pulmão, medula óssea, córnea e osso. O registro inclui também capítulos de transplante pediátrico, informações sobre lista de espera e doação de órgãos. Seus relatórios são publicados trimestralmente com dados parciais e anualmente com dados completos. Tais publicações têm um papel crucial em decisões estratégicas sobre transplante e órgãos pelo governo brasileiro. Atualmente, todas as equipes de transplante cardíaco participam voluntariamente do RBT que inclui 11 variáveis: sexo, idade e tipo doador, identidade do receptor, sexo do receptor, idade do receptor, data da cirurgia de transplante, data da perda do enxerto (se aplicável), causa da perda de enxerto (se aplicável), data do último acompanhamento, data e causa de morte (se aplicável). O RBT fornece um selo de qualidade aos centros que tiverem obtido uma taxa de submissão de 100%. O comprometimento ao banco de dados por parte dos centros de transplante cardíaco é heterogêneo. No ano passado, somente 43% dos centros de transplante cardíaco receberam um selo de qualidade, o que representa 50% de dados faltantes de pacientes considerando o total de transplantes cardíacos realizados. Em 2023, foram realizados 424 transplantes no Brasil, em 13 estados por 48 equipes de transplante.^2^ Isso foi um grande feito considerando o período inicial de transplante de órgãos sólidos. A Figura 1 mostra o aumento progressivo de transplante cardíaco no Brasil; 2023 foi um marco no número de transplantes cardíacos (Figura 2), bem como no número de doações efetivas de órgãos realizadas no Brasil.

Não há dúvidas de que precisamos melhorar o banco de dados de transplante cardíaco no RBT. Trata-se de uma velha demanda de nossa comunidade de transplante, a Sociedade Brasileira de Cardiologia^3^ e a Sociedade Brasileira de Cirurgia Cardiovascular. Isso nos dará uma informação mais clara e significativa sobre as atividades e desfechos de transplante cardíaco em nosso país, para promover iniciativas de melhoria de qualidade e para discutir novos sistemas de reembolso aos usuários do sistema de saúde público e privado que contribuirão para a sustentabilidade financeira de nossos programas. A participação de centros brasileiros de transplante torácico no registro ISHLT pode ser crucial para o avanço em nosso registro. As lideranças da ABTO e da ISHLT já assinaram um acordo de compartilhamento de dados do registro. Isso significa que todos os centros de transplante continuarão a preencher o RBT, que está em português, com seus próprios dados de maneira *online*. Uma vez ao ano, a ABTO irá transferir todos os dados em inglês para o registro ISHLT de maneira segura e anônima, sem identificação de pacientes e/ou hospitais. Isso nos dará a oportunidade de participar ativamente em um banco de dados global muito importante, no qual teremos padrões e referências internacionais de desfecho para comparar. Finalmente, isso abre a oportunidade para pesquisa e educação com foco em nossas necessidades nacionais.

Nesse sentido, o RBT foi modificado em uma nova plataforma, versão 2.0. Isso foi particularmente importante para alcançar padrões legais de proteção, confidencialidade, privacidade dos dados, e integridade da informação. Durante esse período de modernização de nosso registro, novas variáveis foram incluídas com as mesmas codificações do registro ISHLT, o que facilitará o processo de exportação de dados. Uma vez que o conjunto completo de dados do registro ISHLT possui mais de 100 variáveis, seria prático adicionar neste momento somente nove variáveis além daquelas incluídas na versão original da ABTO e progressivamente incorporar novas variáveis.

Para o sucesso da parceria / acordo entre a ABTO e a ISHLT, é fundamental que todos os centros brasileiros de transplante cardíaco participem ativamente no registro. Uma conscientização universal sobre a importância do registro é extremamente relevante para nossos pacientes para todos nós
