# Supplementary material for: Registro Brasileiro de Transplantes da ABTO e Registro de Transplante Cardíaco da ISHLT: Uma Valiosa Parceria
Source: Arq Bras Cardiol. 2025 Feb 4;122(1):e20240370. [Article in Portuguese] doi: 10.36660/abc.20240370 (PMC11809913; doi:10.36660/abc.20240370)
Supplement: Translation

# Translation: ABTO Brazilian Transplant Registry and ISHLT Heart Transplant Registry: An Important/Valuable Partnership

**DOI:** 10.36660/abc.20240370i

**Authors:** Fernando A. Atik, Paulo Manoel Pego-Fernandes, Juan A. Mejia, Livia A. Goldraich, Fabiana G. Marcondes-Braga, Estela Azeka, Fernando Augusto Figueira, Valter Duro Garcia, Luciana Haddad, Wida Cherikh, Josef Stehlik, Rebecca Cogswell

**Affiliations:** 1 Brasília DF Brazil Universidade de Brasília, Brasília, DF – Brazil; 2 São Paulo SP Brazil Instituto do Coração do Hospital das Clínicas da Faculdade de Medicina da Universidade de São Paulo, São Paulo, SP – Brazil; 3 Fortaleza CE Brazil Hospital de Messejana, Fortaleza, CE – Brazil; 4 Porto Alegre RS Brazil Hospital de Clínicas de Porto Alegre – Serviço de Cardiologia, Porto Alegre, RS – Brazil; 5 Recife PE Brazil Instituto de Medicina Integral Professor Fernando Figueira – Cardiologia Adulto, Recife, PE – Brazil; 6 Porto Alegre RS Brazil Santa Casa de Misericórdia de Porto Alegre, Porto Alegre, RS – Brazil; 7 Richmond Virginia USA United Network for Organ Sharing, Richmond, Virginia – USA; 8 Salt Lake City Utah USA The University of Utah School of Medicine – Division of Cardiovascular Medicine, Salt Lake City, Utah – USA; 9 Minnesota EUA University of Minnesota – Department of Medicine, Division of Cardiology Minneapolis, Minnesota – EUA

**Keywords:** Records, Transplantation, Heart Transplantation

Since 1983, data of more than 220,000 heart, lung and heart-lung transplant recipients from 481 heart transplant centers and 260 lung transplant centers in 47 countries have been submitted to the International Society for Heart and Lung Transplantation (ISHLT) Registry.^1^ The ISHLT Registry mission is to improve the care of patients with advanced heart and lung disease. The ISHLT Registry has engaged members from diverse regions, provided outcome data from thoracic transplants worldwide and catalyzed scientific and clinical collaboration. Ten Brazilian hospitals used to participate in the ISHLT Registry in the past. During this period, some problems were observed. First, there was heterogenous commitment among participating centers to the complete dataset. Second, there was lack of human resources in some hospitals dedicated to the Registry. Third, data collection was each center's responsibility and submission of the data to the ISHLT registry was not mandatory.^1^

In 2021, the ISHLT Registry was interrupted due to new data sharing regulations and data collection approaches. Data fields and data upload process were updated. In 2024, the ISHLT Registry began to exchange data only with national and regional registries, such as United Network for Organ Sharing and Eurotransplant, and no longer with individual hospitals or institutions.

In order for Brazil to continue its participation in the new ISHLT Registry format, it was understandable that the Brazilian Association for Organ Transplantation (ABTO) was chosen as the national representative society since it had already established its own registry, The Brazilian Transplant Registry. The Brazilian Transplant Registry started in 1996, and it is the official report of all transplants performed in Brazil, including kidney, liver, heart, pancreas, lung, bone marrow, cornea and bone. It also includes chapters on pediatric transplantation, information about waiting list and organ donation. Its reports are published quarterly and yearly, with partial and complete data, respectively. These publications have a crucial role in strategic decisions on organ transplantation by the Brazilian government. Currently, all heart transplant teams participate voluntarily in the Brazilian Transplant Registry which includes 11 variables: donor sex, donor age, type of donor, recipient identification, recipient sex, recipient age, date of transplant surgery, date of graft loss (if applicable), cause of graft loss (if applicable), date of last follow-up, date and cause of death (if applicable). The Brazilian Transplant Registry provides a quality label for those centers that have had 100% data submission rate. Commitment to the database is heterogeneous among heart transplant centers. Last year, only 43% of heart transplant centers received a quality label, that represents 50% patient missing data regarding the total number of heart transplants performed. In 2023, 424 heart transplants were performed in Brazil, in 13 states by 48 transplant teams.^2^ That has been a huge achievement since the early period of solid organ transplantation. Figure 1 shows the progressive increase of heart transplantation in Brazil; 2023 was a milestone in the number of heart transplants (Figure 2), as well as in the number of effective organ donations performed in Brazil.

**Figure 1:** Number of heart transplants in Brazil by decade from 1995 to 2023.

**Figure 2:** Number of heart transplants in Brazil by decade from 1995 to 2023.

There is no question that we need to improve the heart transplant database within the Brazilian Transplant Registry. This is an old demand of our transplant community, the Brazilian Society of Cardiology^3^ and the Brazilian Society of Cardiovascular Surgery. That will give us a clearer and meaningful information about heart transplant activities and outcomes in our country, to promote quality improvement initiatives and to discuss new reimbursement systems to public and private health users that will contribute to the financial sustainability of our programs. The participation of Brazilian thoracic transplant centers in the ISHLT Registry may be key to advance our own registry. Both the ABTO and the ISHLT leadership have already signed a Registry Data Sharing Agreement. That means that all transplant centers will continue to fill their own data in the web-based Brazilian Transplant Registry that is in Portuguese. Once a year, ABTO will transfer all data in English to the ISHLT Registry securely, anonymously, with no identification of patients and/or hospitals. That will provide us the opportunity to actively participate in a very relevant global database, in which we will have international outcome standards/benchmarks to compare. Finally, it opens the opportunity for education and research, focused on our national needs.

In this regard, the Brazilian Transplant Registry was modified into a new platform version 2.0. That was particularly important to reach legal standards of data protection, confidentiality, privacy and information's integrity. During this period of modernization of our Registry, new variables were included with the same codifications of the ISHLT Registry, which will facilitate the data exporting process. Since the ISHLT Registry complete dataset has more than 100 variables, it would be practical to add at this point only nine variables in addition to those included in the ABTO original version and progressively incorporate new ones.

In order for the ABTO and ISHLT partnership/agreement to be successful, it is fundamental that all Brazilian heart transplant centers actively participate in the Registry. Universal awareness of the importance of the Registry is extremely relevant for our patients and for all of us!
